# Transcriptome response of cassava leaves under natural shade

**DOI:** 10.1038/srep31673

**Published:** 2016-08-19

**Authors:** Zehong Ding, Yang Zhang, Yi Xiao, Fangfang Liu, Minghui Wang, Xinguang Zhu, Peng Liu, Qi Sun, Wenquan Wang, Ming Peng, Tom Brutnell, Pinghua Li

**Affiliations:** 1 Key Laboratory of Biology and Genetic Resources of Tropical Crops, The Institute of Tropical Bioscience and Biotechnology (ITBB), Chinese Academy of Tropical Agricultural Sciences (CATAS), Haikou, Hainan 571101, China; 2 Center for Plant Science Innovation, University of Nebraska-Lincoln, Lincoln, Nebraska 68583, USA; 3 Department of Agronomy and Horticulture, University of Nebraska-Lincoln, Lincoln, Nebraska 68583, USA; 4 CAS-Key laboratory of Computational Biology, CAS-MPG Partner Institute for Computational Biology, Shanghai Institutes for Biological Sciences, CAS, Shanghai 200031, China; 5 Department of Statistics, Iowa State University, Ames, Iowa 50011, USA; 6 Computational Biology Service Unit, Life Sciences Core Laboratories Center, Cornell University, Ithaca, New York 14850, USA; 7 Donald Danforth Plant Science Center, St. Louis, Missouri 63132, USA; 8 State Key Laboratory of Crop Biology, College of Agronomic Sciences, Shandong Agricultural University, Tai’an, Shandong 271018, China

## Abstract

Cassava is an important staple crop in tropical and sub-tropical areas. As a common farming practice, cassava is usually cultivated intercropping with other crops and subjected to various degrees of shading, which causes reduced productivity. Herein, a comparative transcriptomic analysis was performed on a series of developmental cassava leaves under both full sunlight and natural shade conditions. Gene expression profiles of these two conditions exhibited similar developmental transitions, e.g. genes related to cell wall and basic cellular metabolism were highly expressed in immature leaves, genes involved in lipid metabolism and tetrapyrrole synthesis were highly expressed during the transition stages, and genes related to photosynthesis and carbohydrates metabolism were highly expressed in mature leaves. Compared with the control, shade significantly induced the expression of genes involved in light reaction of photosynthesis, light signaling and DNA synthesis/chromatin structure; however, the genes related to anthocyanins biosynthesis, heat shock, calvin cycle, glycolysis, TCA cycle, mitochondrial electron transport, and starch and sucrose metabolisms were dramatically depressed. Moreover, the shade also influenced the expression of hormone-related genes and transcriptional factors. The findings would improve our understanding of molecular mechanisms of shade response, and shed light on pathways associated with shade-avoidance syndrome for cassava improvement.

Cassava (*Manihot esculenta* Crantz) is the most important tropical root crop globally and provides staple food for over 700 million people, particularly in developing countries in Africa (51%), Asia (29%) and South America (20%)^1^. Since it is well adapted to barren soil and drought conditions and its storage roots are enriched in starch^2^, cassava is widely considered as an important food reserve to battle global famine^3^. With the ongoing climate change and looming global energy crisis, cassava is also an ideal crop for bioenergy generation, biomaterial production and animal feed due to its high biomass productivity and high starch quantity and quality^4^.

In the humid and sub-humid tropical areas in Latin America and Africa, cassava is usually cultivated intercropping with grain crops such as maize, rice or grain legumes (beans, cowpeas or groundnuts) that mature earlier^5^. It could also be intercropped with perennial crops such as oil palm, rubber and coconut trees for maximum land economy^6^,^7^. In the cases where the associated crop grows faster, for example in the most dominated intercropping combination with maize, cassava is always subjected to certain degrees of shading especially during their early stages of development, thus its productivity is drastically reduced^8^,^9^. Besides, long cloudy periods, a typical climate feature in humid tropics^10^, would also cause low light intensity of canopy illumination, hence, lead to lower yield in these regions. Thus study the shade response of cassava is very important for both intercropping management and genetic improvement.

Under a leaf canopy (shade condition), both light quality and intensity could be changed, more specifically, relatively higher far-red (FR) but lower red (R) and blue light^11^. Both R:FR ratio and blue light, as well as the total light intensity, are signals that can induce shade-avoidance syndrome (SAS)^12^,^13^ that causes the elongation of stems and petioles. Plants grow tall and spindly for sunlight competition at the expense of leaves development, and finally the yield was reduced^14^. The ultimate goal is to eliminate/minimize SAS in crops. There are evidences that in maize and tobacco, yield can be increased if the shade avoidance behavior can be suppressed^14^,^15^.

The phytochromes (PhyA-phyE in *Arabidopsis*), the receptors of R and FR radiation, play a key role in inducing the shade avoidance syndrome of plants^16^. Phytochromes are photochromic biliproteins that exist in two convertible isoforms: Pr and Pfr. Pr is the inactive form, which can be converted to the active form of Pfr when absorbing the red light; on the other hand the active form of Pfr can switch back to the inactive form of Pr after absorption of FR light^17^. The Pfr form translocates to the nucleus under normal sunlight, where it binds and degrades a subset of basic-helix-loop-helix (bHLH) transcription factors (PIFs). Under shade condition with low R:FR ratio, the pool of PIFs increases, the PIFs bind promoters and regulate the expression of genes that promote the shade avoidance, including the homeodomain-Leucine zipper (HD-Zip) II family members, e.g. AtHB-2 and AtHB-4 in *Arabidopsis*, as well as genes that play critical roles in auxin biosynthesis, signaling and transport^17^,^18^,^19^. Recently, ethylene was also reported to promote a light quality-mediated SAS in *Arabidopsis*^20^,^21^. The molecular mechanism of shade avoidance is emerging in recent years; however, these studies were mainly conducted in model plant of *Arabidopsis*, while much less information is available for food crops, especially for tropical crops (e.g., cassava). Therefore, it would be important to increase our understanding of the molecular mechanisms of shade response in cassava, which is directly associated with its production when intercropping with other crops and/or under cloudy weather.

With the rapid development of molecular biotechnologies and bioinformatics, large-scale gene expression analysis has become available in cassava, e.g. EST and cDNA libraries have been sequenced to identify genes responsible for economically important traits such as starch content, disease and stress resistance^22^,^23^,^24^,^25^ and cDNA and oligonucleotide microarray have been used to identify differential expressed genes associated with post-harvest physiological deterioration^26^, bacterial blight disease^27^, storage roots development^28^, as well as the response to cold and drought stresses^29^,^30^. Besides, the release of draft genome (http://www.phytozome.net/cassava) and rapid improvement of next generation sequencing technologies, e.g. Illumina RNA-seq, which overcome the shortcomings of microarray including high levels of noise and cross-hybridization among gene family members *et al*.^31^, open a new way to study the gene expression on global level in cassava research.

In this study, we used a natural shade environment simulating the intercropping system in the field, to investigate the gene expression dynamics of cassava leaves under different canopy shading levels using RNA-seq technology. Our objectives are: (1) to examine the gene expression profiling at different stages of leaf development under full sunlight (control) and natural shade conditions, respectively; and (2) to compare the differential gene expression between shade and control. The results will provide new insights into the shade response of cassava, and improve our understanding of mechanisms that trigger SAS and help cassava improvement of the yield.

## Results

### Growth response of cassava to the natural shade

The uniform cassava seedlings were planted under full sunlight (control) and natural shade condition (under the rubber tree seedlings, Supplemental Figure S1). The plants under the two conditions were about 100 meters apart, to ensure similar environmental temperature and humidity. The main variable in this study, environmental light (including light spectrum and intensity), was monitored periodically. Compared with the control, light intensity under the shade was drastically reduced, with the ratio between the two conditions varied from 0.05 in sunny days to 0.27 in cloudy days. As expected, the red (R) to far-red (FR) light ratio was decreased from 2.7 in control to 0.8 in shade condition (Supplemental Figure S2).

**Figure 1:**
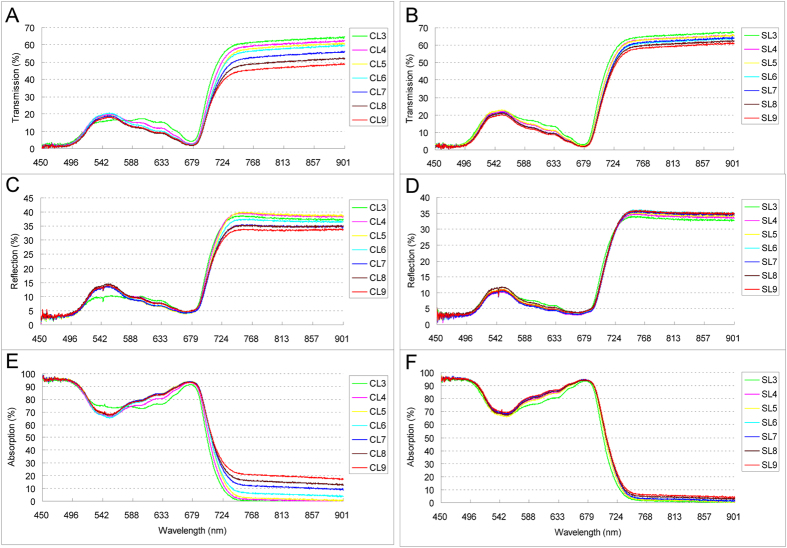
Light spectrum of cassava expanded leaves. The curves represent transmission (**A,B**), reflection (**C,D**) and absorption (**E,F**) percentage of expanded leaves in both natural (control) and shade conditions, respectively. Leaves from young to old: CL3-CL9 for control and SL3-SL9 for shade.

Transmission and reflection spectra were measured on fully expanded leaves including from the third (L3) to the ninth leaf (L9) counting from top to bottom of the canopy (Fig. 1). In average, ~82.9% of visible light (450~700 nm) was absorbed by cassava leaves, while ~9.8% was transmitted and ~7.3% was reflected. The ratios were very similar between the two conditions (Fig. 1). Interestingly, in the far-red and infra-red range (700~900 nm), the percentage of transmission and reflection substantially decreased but absorption increased in control as leaf grew from young to old. However, this trend is much weaker under the shade condition (Fig. 1). In addition, the photosynthetic parameters such as Pn (net photosynthetic rate) under the shade was significantly decreased, while Gs (stomata conductance), E (transpiration rate) and Ci (intercellular CO2 concentration) were slightly increased, comparing to the control (Supplemental Figure S3). Moreover, the first fully expanded leaf (L3) had the least Pn and E and Gs, while no significant change was observed from leaf four to nine (L4-L9) in both conditions.

**Figure 2:**
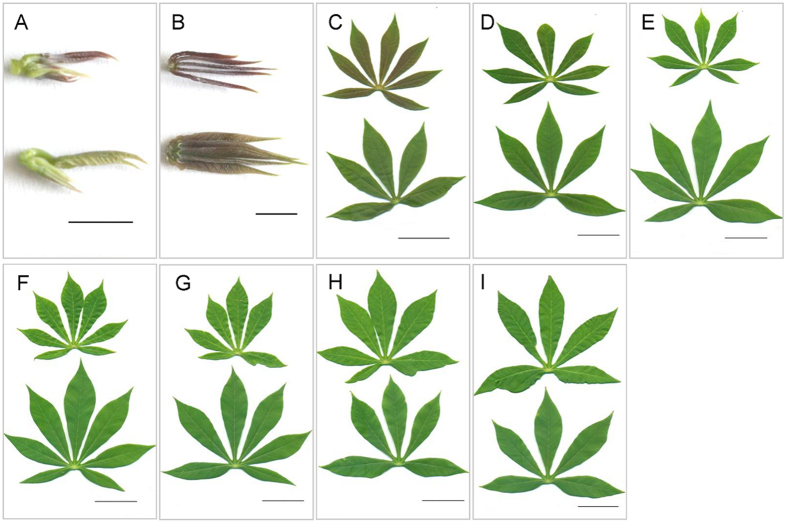
Samples of cassava leaves used in this study. In each graph, sample in the top was derived from control, while sample in the bottom was derived from shade. A-I represent eighteen RNA-seq leaf samples (CL1-CL9 vs. SL1-SL9) used in this study respectively. Bars represent 0.5 cm (**A,B**) and 5 cm (**C–I**), respectively.

Morphologically, seedlings grown under shade have greatly increased leaf size, petiole length, plant height, leaf number and more chlorophyll accumulation but smaller stem diameter in shaded seedlings compared with the control (Fig. 2 and Supplemental Figure S4). We noticed that the higher plant height under the shade condition than the control was mainly due to the fast elongation of the internodes in the middle-upper part of shaded seedlings.

### Transcriptome changes during the leaves development

In order to investigate how the transcriptome change during leaf development, we included two un-fully expanded leaves (L1 and L2) besides the fully expand leaves L3 to L9 in our RNA-seq analysis. Leaves at different stages are shown in Fig. 2. Leaf one (L1) included a ball of unexpanded leaves and an expanding leaf with no developed leaf lobes. Leaf two (L2) has developed leaf lobes that had not fully expanded. Three biological replicates from each layer of leaves were collected from plants under control and shade conditions, with each replicate pooled from six seedlings. A total of 54 libraries were constructed for RNA-seq experiments. 348 million single-end 51 bp reads were generated from all libraries combined. After trimming adapters and filtering out low quality reads, around 306 million reads (~88.2%) were aligned to the cassava genome and used for further analysis. Overall, the genic distribution of reads showed that most reads (76%) were mapped to exon/protein coding region, while the others were distributed among intron (1%) and intergenic (9%) region as well as splicing junction (14%) region (Supplemental Figure S5). The genic distribution of reads was similar in samples grown under shade and control (sunlight) conditions. Gene expression was detected in 54632 transcripts, which corresponding to 32439 loci, from 54 samples using Cufflinks. Of these transcripts, 33.7% (18403) were potentially novel isoforms based on novel splicing junctions identified, which corresponding to 9827 reference loci. In addition, 3018 intergenic transcripts (5.5% of total transcripts), which were not annotated in cassava reference genome, were identified. Using a machine learning method (random forest) to eliminate the background noise of these unknown transcripts, 2465 were predicted as real transcripts (AUC = 0.974, see Methods). Among them, 59 were protein coding transcripts and 2294 were non-coding ones (Supplemental Table S1). Interestingly, we noticed an increase of splicing junctions along the leaf development in both shade and control conditions, which may indicate that the older leaves developed more complex transcript variants than younger leaves (Supplemental Figure S6). There was no major difference in transcript variant between shade and control, with 73.2% genes have only one transcript, 16.2% have two variants, and only 1.1% of genes have greater than five variants (Supplemental Figure S7).

An arbitrary threshold cutoff, FPKM >1, was used to identify genes expressed in this study. In total, 23125 genes were expressed in the cassava leaves from 54 samples, which equal to 75.4% of annotated genes in the genome (phytozome Mesculenta v4.1). Among them, 21994 (95.1%) were expressed in both shade and control samples, whereas only a few genes (665 vs. 466) were expressed exclusively in one growth condition. Comparison among samples in each canopy layer showed that 16356 genes were expressed in all shade samples and 17050 in all control samples. Younger leaves (L1 and L2) expressed more genes than older leaves (L3 to L9, Supplemental Figure S8). We also used qRT-PCR to validate the expression levels of ten genes, and the result revealed a high correlation (r = 0.88) between RNA-seq and qRT-PCR (Supplemental Table S2).

### Developmental dynamics of cassava leaves

In order to better understand the gene expression changes along leaf development, two methods were applied to identify differentially expressed (DE) genes using 23125 genes that were expressed in both shade and control conditions. First, we used pairwise comparison implemented in Cuffdiff program (FDR <0.001) to identify the DE genes that up or down regulated across the leaf samples in shade and control conditions, respectively (Supplemental Figure S9). Interestingly, we noticed the transcriptome of leaf one and two were dramatically different from other leaves, since more DE genes were identified when other leaves compared with L1 or L2 in both shade and control conditions (Supplemental Figure S9). The union of 9020 and 11389 DE genes identified by pair-wise comparison presents the DE genes across the samples in control and shade conditions, respectively. Secondly, we used the overall test implemented in DESeq to identify DE genes across all samples with FDR <0.001 in control and shade as well. The overlapping genes between two methods, 8197 in control and 10779 in shade (Supplemental Figure S10), were considered as strictly regulated by the development. Of these genes, 7274 (67.5% of DE genes in shade and 88.7% in control) were shared between control and shade conditions (Supplemental Figure S10), which indicate genes involved in leaf development were similar between two growth conditions.

**Figure 3:**
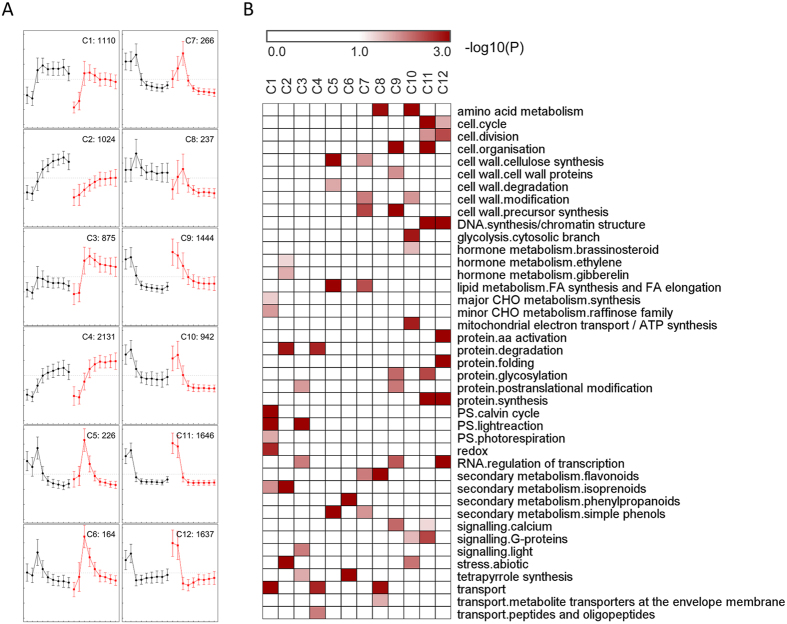
Dynamic transcriptome of cassava leaves. (**A**) Expression patterns of 12 clusters along different developmental leaves. The samples are (from left to right): CL1-CL9 in black for control, and SL1-SL9 in red for shade conditions, respectively. Error bars represent standard deviation. The number of genes included in each cluster is shown at the upper-right corner. (**B**) Functional category enrichment of each cluster in (**A**).

To further explore the biological function of these DE genes during leaf development and exam the influence of shade on their expression, a total of 11702 genes representing the union of DE genes between control and shade were clustered with self-organization tree algorithm using Pearson correlation, and MapMan annotation was used to assign genes into functional categories. A total of 12 clusters were identified (C1-C12, Fig. 3) and functional category enrichment analysis was performed for each cluster to identify the pathways divergence during leaf development under control and shade conditions.

As shown in Fig. 3A, C1 to C4 clusters included genes that had increased expression from immature (L1 and L2) to mature leaf (L3 to L9). The genes in C1 cluster had low expression in L1 and L2, however, their expression was constantly higher from L3 to L9 in both control and shade conditions. The most significantly enriched functional categories in this cluster were Calvin cycle, light reactions and photorespiration of photosynthesis, followed by carbohydrate metabolism, secondary metabolism of isoprenoids, redox, and transport related genes (Fig. 3B). The expression of genes in C2 cluster was influenced by shade, since the gene expression levels in mature leaves were lower in shaded condition than that in control. The enriched categories in this cluster included hormone metabolism related genes, e.g. genes involved in ethylene and gibberellin metabolism, followed by abiotic stress, protein degradation and secondary metabolism of isoprenoids. The C3 cluster had similar expression pattern as C1, however, the genes in this cluster had higher expression in shade than that in control in mature leaves (L3 to L9). The enriched categories in this cluster included protein modification related genes, as well as genes involved in light reaction of photosynthesis, RNA regulation of transcription, light signaling, and tetrapyrrole biosynthesis. The expression of genes in cluster C4 was gradually increased from L1 to L9, which enriched genes that annotated to play a role in peptides transport and protein degradation.

The genes in C5 to C8 clusters had peak expression in L3, which was the first fully expanded leaf counted from top to the bottom of canopy. The enriched genes included those that are required for secondary cell wall biosynthesis (e.g. cellulose synthesis, FA synthesis and FA elongation of lipid), tetrapyrrole biosynthesis, secondary metabolism of flavonoids, phenylpropanoids and simple phenols, and metabolite transporters at the envelope membrane, suggesting a major reprogramming of the leaf transcriptome as the leaf builds its photosynthetic machinery.

The genes from C9 to C12 clusters had higher expression in the immature leaves (L1 and L2) than that in mature leaves (L3 to L9). The expression of genes in C9 and C10 clusters was gradually decreased from L2 to L3, however, sharply decreased from L2 to L3 in cluster C11 and C12. The enriched categories in these clusters indicate an active metabolism in immature leaves, since the genes in these clusters encoding enzymes for cell wall biosynthesis (C9 and C10), cell cycle, and cell division (C11 and C12), cell organization regulation (C9 and C11), DNA synthesis and chromatin structure (C11 and C12), amino acid metabolism (C10), protein metabolism (C9, C11 and C12), signaling regulation of calcium (C9 and C11) and G-proteins (C10 and C11), brassinosteroid biosynthesis and signaling (C10), respiratory pathways (glycolysis, mitochondrial electron transport/ATP synthesis, C10). Similar results were also obtained from GO term enrichment analysis (Supplemental Table S3). Taken together, the DE genes in C1 to C9 clusters reveal that the major biochemical shifts along the leaf development are produced in part by highly dynamic, coordinated and localized transitions in mRNA abundance, and shade affected gene expression during leaf development.

### Transcriptome in response to natural shade

Based on physiological measurement (e.g. Pn, E and Ci) and phenotypic observation (e.g. leaf shape and size), we concluded that leaf 6 (L6) to 9 (L9) were in the mature stage. The high similarity among L6 to L9 presented by principal component analysis (PCA, Supplemental Figure S11) and small number of DE genes presented by pair-wise comparison (Supplemental Figure S9) in either shade or control conditions indicated that L6-L9 leaves were developmentally identical and the difference of gene expression of matured leaves grown in shade and control conditions was mainly caused by shade. So that we can compare the transcriptomes directly between shade and control by using pooled data from those matured leaves (L6 to L9) to explore genes affected by natural shade.

**Figure 4:**
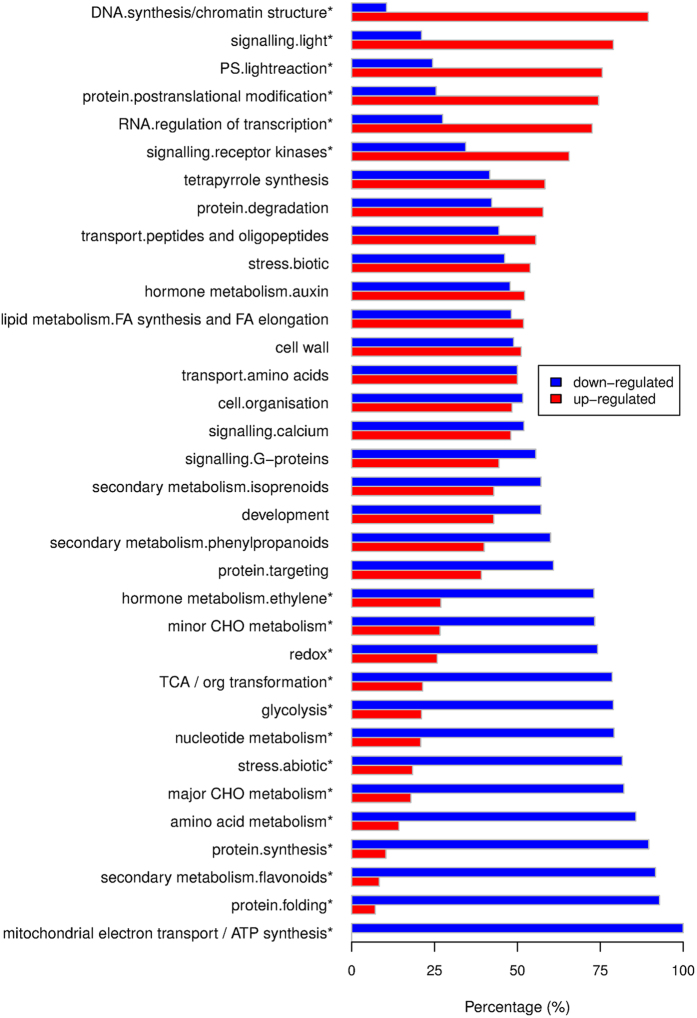
Percentage of up- and down- regulated genes in shade compared with control. The categories were derived from MapMan annotation. Star (*) indicates p < 0.05 based on binomial test.

In total, 2881 DE genes were identified between shade and control conditions (Supplemental Table S4). As shown in Fig. 4, many pathways were greatly induced by shade, e.g. DNA synthesis/chromatin structure, light signaling, light reaction of photosynthesis and RNA regulation of transcription. On the contrary, the major CHO (carbohydrate) catabolic pathways including mitochondrial electron transport, TCA and glycolsis, as well as secondary metabolism, amino acid metabolism, protein synthesis and folding, redox and abiotic stress were significantly depressed. In addition, genes in some pathways showed a binary change pattern that half DE genes were up-regulated while another half DE genes were down-regulated, e.g., auxin, FA synthesis and FA elongation, cell wall and cell organization (Fig. 4). To further characterize the individual genes whose expression was influenced by the natural shade, heatmap was used to represent the expression of DE genes that were involved in those pathways.

### Shade induced pathways

**Figure 5:**
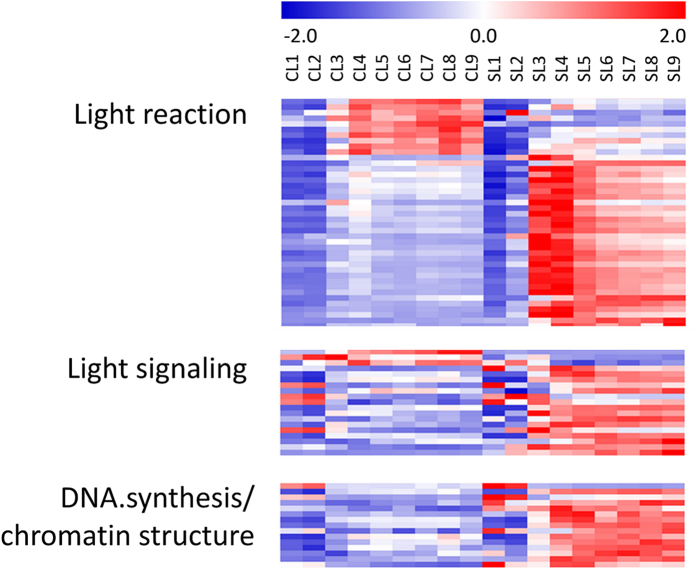
Heatmap of the genes related to light reaction, light signaling and DNA synthesis/chromatin structure.

We noticed that shade mainly affects light reaction in cassava leaves. In total 51 photosynthesis related genes that differentially expressed between shade and control, 80% (41/51) of them were involved in light reaction, e.g. PSI polypeptide subunits of PSAD, PSAE, PSAF, PSAN, PSAO and PSAK; PSII polypeptide subunits PSBO, PSBP, PSBQ, PSBW and PSBX; as well as light harvesting complex (LHC) genes. Although a few of them were depressed by shade, most of them were highly induced by shade (Fig. 5).

Similar to light reaction of photosynthesis, most light signaling related genes were also greatly induced by natural shade (Fig. 5 and Supplemental Table S4), including phototropin light receptor PHOT1, NPH3 (Non-phototropic hypocotyl 3) family members that function as signal transducer in phototropism, blue light photoreceptor CRY2 and PAS/LOV protein B; and red/far-red signaling related genes such as phytochrome kinase substrate (PKS1 and PKS2) and SPA family protein (SPA3, suppressor of phyA-105, Supplemental Table S4).

Likewise, genes associated with DNA synthesis/chromatin structure, e.g., sister chromatid cohesion 1 protein 4 (SYN4) and telomeric DNA binding protein 1 (TBP1) were induced by shade (Fig. 5 and Supplemental Table S4). We also observed the induction of nitrogen metabolism, since several important genes, e.g., nitrate reductase (NIA), glutamate synthase (NADH-GOGAT) and glutamate dehydrogenase (GDH), were significantly induced by shade (Supplemental Table S4).

### Shade depressed pathways

**Figure 6:**
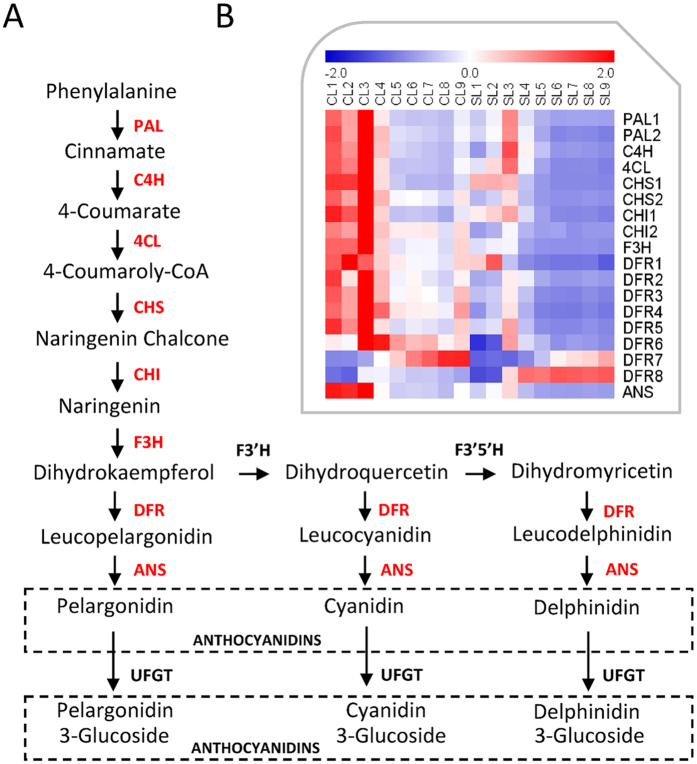
Anthocyanin biosynthesis pathway (**A**) and heatmap of its related genes (**B**). Anthocyanin biosynthesis pathway was modified from Gou *et al*. (2011) and Petroni and Tonelli (2011).

We observed that younger leaves grown in control condition accumulated more purple pigments than that of shaded plants, and then we further examined the anthocyanins biosynthesis pathways, which may help protect leaves from high solar exposure and ultraviolet radiation. Amazingly, we found that all enzymes that involved in the entire anthocyanins biosynthesis pathways were depressed in shaded leaves (Fig. 6). In addition, we observed that most anthocyanins biosynthesis related genes were highly expressed in the unexpanded leaves (L1 and L2), reached their highest expression levels in the first expanded leaves (L3), and then decreased from L4 to L9, which was highly consistent with the distribution of purple color among leaves (Fig. 2). This suggested that purple pigments accumulation in cassava leaves under high light was mainly derived from anthocyanins biosynthesis, and anthocyanins might play an important role in protecting younger leaves from radiation.

**Figure 7:**
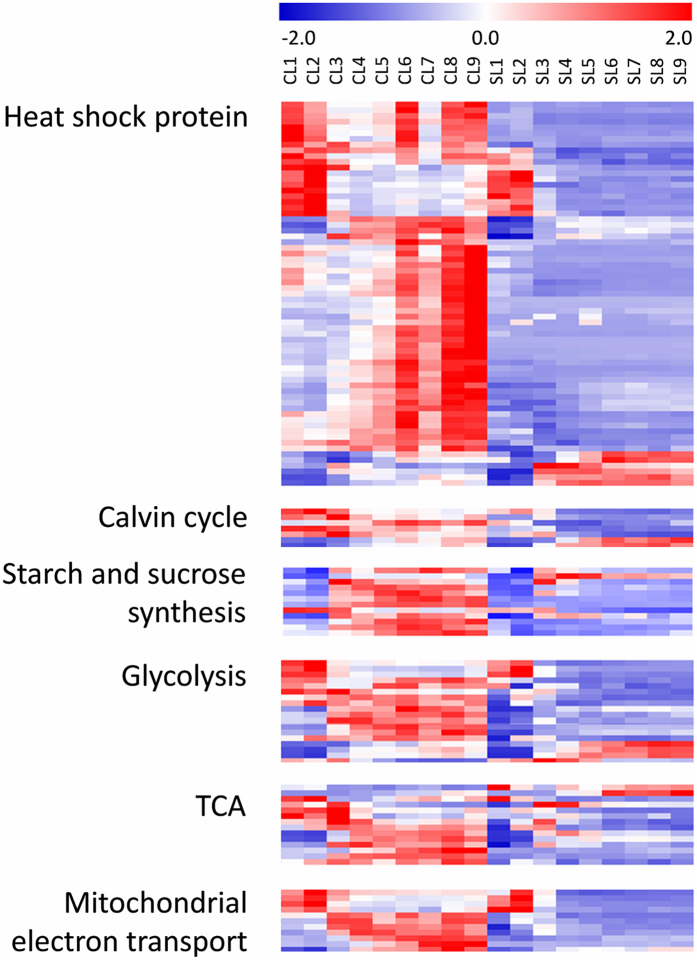
Heatmap of the genes associated with heat shock protein, calvin cycle, starch and sucrose synthesis, glycolysis, TCA and mitochondrial electron transport.

Consistent with anthocyanin biosynthesis, heat shock proteins (HSP) were also dramatically repressed in the shaded condition (Fig. 7). HSP families, including HSP15, HSP17-18, HSP21-23, HSP26, HSP70, HSP81 and HSP90, were significantly down-regulated from L1 to L9 in shade while up-regulated in control (Supplemental Table S4), which may indicate less oxidative stress and temperature under shaded condition. We do notice that the expression of redox scavenging related genes, e.g. ascorbate peroxidase and superoxide dismutase, was significantly lower in shaded leaves than that in control (Supplemental Table S4).

Genes associated with calvin cycle were also expressed lower in shaded leaves. It is worth to note that, the expression of four out of five fructose-bisphosphate aldolase (FBA), which catalyzes the last reversible reaction of calvin cycle from triose phosphates dihydroxyacetone phosphate (DHAP) and glyceraldehyde 3-phosphate (GAP) into fructose 1,6-bisphosphate, was dramatically depressed by shade (Fig. 7 and Supplemental Table S4). Associate with calvin cycle, the expression of starch and sucrose biosynthesis-related genes was also depressed by shade. As shown in Fig. 7, the shade dramatically decreased the expression of small and large subunits of AGPase (ADP-glucose pyrophosphorylase), as well as starch synthase (SS) and starch branching enzymes (SBEs), which participated in the synthesis of starch metabolism. Similar changes were observed in the sucrose synthetic related genes such as sucrose-phosphate synthase (SPS), sucrose-phosphatase (SPP) and sucrose transporters (SUT). Consistent with starch and sucrose biosynthetic genes, the expression of genes encoding enzymes in galactinol and raffinose synthases, which catalyses the first and second steps of RFOs (raffinose family of oligosaccharides) biosynthesis, was also repressed by shade (Supplemental Table S4).

Genes involved in respiratory metabolism were influenced by shade as well. As shown in Fig. 7, the enzymes associated with cytosolic branch of glycolysis including enolase, glyceraldehyde 3-phosphate dehydrogenase (GAP-DH), phospho-enol-pyruvate carboxylase (PEPC), pyruvate kinase (PK) and UDP-glucose pyrophosphorylase were all expressed lower in shade than those in control condition (Supplemental Table S4). Similar tendency was also observed for genes in plastid branch of glycolysis, e.g., phosphofructokinase (PFK). Genes involved in TCA cycle, including citrate synthase, NAD-dependent malic enzyme, aconitase, malate dehydrogenase, pyruvate dehydrogenase E3 and succinate dehydrogenase, were all depressed by shade, as well as mitochondrial electron transport/ATP synthesis-related genes (Fig. 7), e.g. cytochrome c, cytochrome c oxidase, cytochrome c reductase, F1-ATPase and the genes encoding NADH dehydrogenase. The low expression of genes related to glycolysis, TCA cycle and mitochondrial electron transport in shade may indicate less energy provision/needed under shaded condition.

### Response of hormone related genes

**Figure 8:**
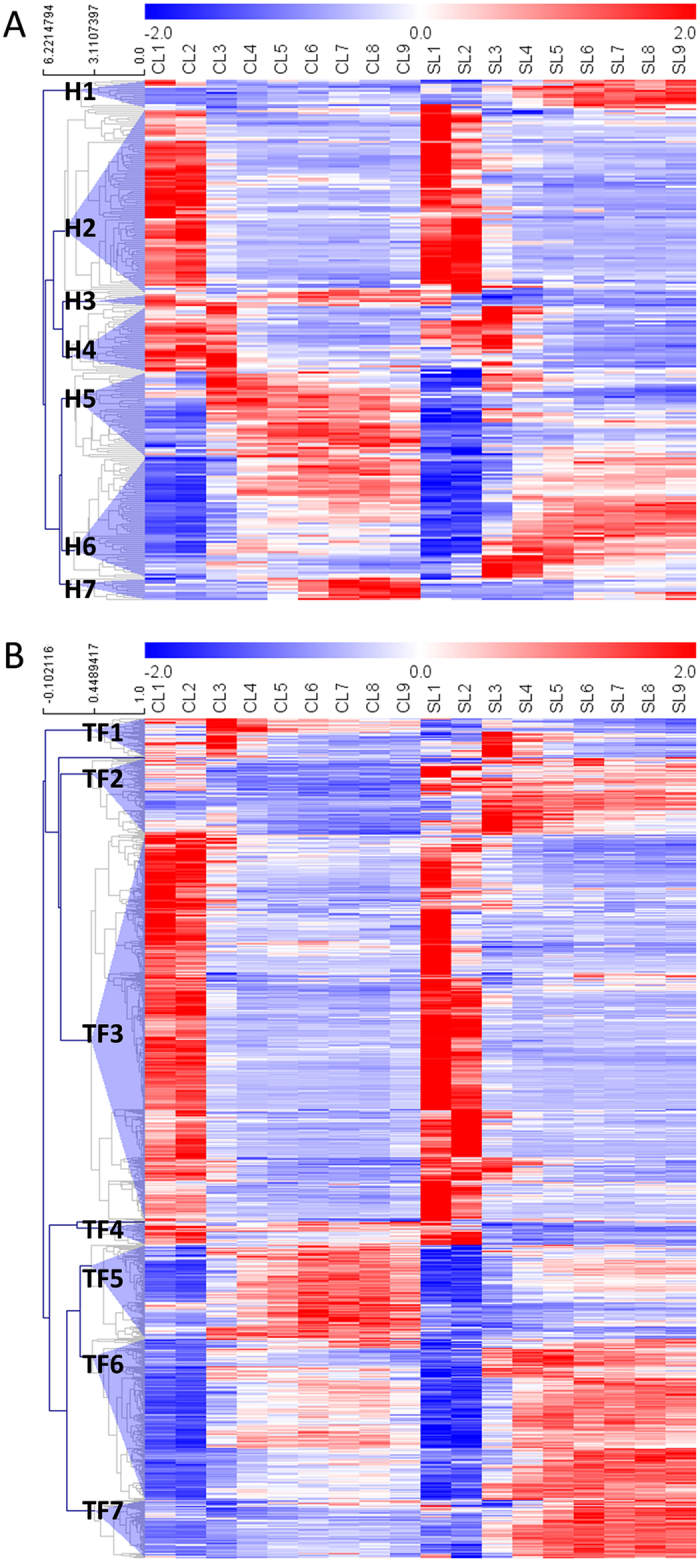
Heatmap of hormone genes (**A**) and transcription factors (**B**) related to leaf development and shade response.

Hormones have been proposed to be essential regulators in plant development. In order to reveal the role of hormones in cassava leaf development and shade response, we monitored the changes of genes involved in the metabolism and signaling of hormones. The 255 hormone related genes (Supplemental Table S5) were grouped into seven clusters based on hierarchical clustering. As shown in Fig. 8A, 91 genes in H2 (hormone 2) cluster expressed highest in immature leaves (L1 and L2) and exhibited similar expression patterns between shade and control conditions. Genes related to ABA (ABF2, ABF3 and ABF5), auxin (PIN1 and PIN3), brassinosteroid (BZR1), ethylene (ERF7 and ERF9) and GA (SPY) signaling were included in this cluster (Supplemental Table S5). Similar to H2, 32 genes in H4 also showed similar expression patterns between shade and control conditions. Besides, they expressed higher in both immature leaves and L3 than that in other leaves (e.g., L4-L9). Several genes associated with anthocyanidins biosynthesis (F3H and FLS) and jasmonate signaling pathways (JAR1, JAZ3, AOS) were included in this cluster (Supplemental Table S5). The results indicated that these genes were associated with leaf development but did not involve in shade response in cassava.

Conversely, 42 genes in H5 and 59 genes in H6 are all expressed lower in immature leaves than that in mature leaves. Moreover, they exhibited different expression trends especially for the mature leaves between shade and control conditions (Fig. 8A). Several genes related to GA biosynthesis (GA20OX1) and its receptors (GID1B and GID1C) were found in H5 cluster, while genes related to ethylene (EFE and ACS10) and jasmonate (LOX1, LOX2 and LOX5) biosynthesis were included in H6 cluster. As compared, only a few genes were grouped in the remained clusters. For example, the expression of 14 genes in H1 was greatly induced, while the expression of 6 genes in H3 and 11 genes in H7 was significantly depressed in mature leaves in response to shade stress (Fig. 8A). The results suggested that these genes played important roles in both leaf development and shade stress.

### Response of transcription factors

Transcriptional factors (TFs) have been well demonstrated as they play important roles in plant growth, development and the response to various environmental stresses. According to the hierarchical clustering, 823 TFs that differentially expressed either among samples or between shade and control conditions were clustered into seven major groups, TF1 to TF7 (Supplemental Table S6). As shown in Fig. 8B, about fifty-percent of genes (380) were grouped into TF3 cluster, and they expressed highest in immature leaves and exhibited similar expression patterns between shade and control conditions, indicating that these TFs were only associated with leaf development but did not response to shade stress in cassava.

On the contrary, TFs in TF5-TF7 clusters were all expressed lower in immature leaves than in mature leaves, most importantly, they showed different response to shade stress (Fig. 8B). For example, the expression of TFs in TF5 was greatly depressed in mature leaves. Several TFs responding to UV-light, e.g., MYB4 and WRKY75, were found in this cluster (Supplemental Table S6). As expected, these TFs that involved in UV-light were also participated in the regulation of anthocyanin biosynthesis, and they showed similar expression patterns as anthocyanin biosynthesis genes as well. However, the TFs in TF6 and TF7 were significantly induced by shade, and many TF family members that involved in light signaling pathways were found. For examples, Psudo ARR family (PRR5), HB family (KNAT3), bHLH family (PIF3, PIL1 and PIL5), and GRAS family (GAI and PAT1) were response to red/far-red light; HB family (HB-1), bHLH family (CIB1) and MYB family (MYB60) were response to blue light. The results suggested that these TFs were involved in both leaf development and shade response in cassava.

TFs in TF2 cluster were also induced by shade (Fig. 8B). Several important light-signaling TFs, e.g., CIB1 to blue light and ANL2 to UV-light, were identified in this cluster. As compared, only 38 and 22 TFs were grouped into TF1 and TF4, respectively. Although no clear expression trends were revealed in TF1, the expression of TFs from TF4 was all significantly depressed by shade in mature leaves (Fig. 8B).

## Discussion

Cassava is not only a dietary staple in much of tropical Africa, but also an important source of starch used in animal feed, alcohol production, plywood, glues and many other products. Due to the common farming practice of intercropping with other crops, cassava was subjected to various degrees of shading, which not only decreases its photosynthetic rate but also triggers the shade avoidance syndrome which further impacts the yield. Hence, it would be important to increase our understanding of the molecular mechanisms that associated with shade response in cassava, and develop strategies to minimize SAS.

Exposed to the shaded environments with higher fraction of far-red in the light spectrum (Supplemental Figure S2), cassava plants intend to grow faster, with more leaves generated and leaf size enlarged (Fig. 2 and Supplemental Figure S4). However, the developmental gradients from unexpanded leaves to fully expanded mature leaves were not influenced by the shading. No matter shade or not, the immature unexpanded leaves (L1 and L2) were enriched in gene activities for basic cellular functions, for example, cell wall biosynthesis, cell cycle, cell organization, and DNA synthesis; while fully expanded mature leaves (L4-L9) were mainly enriched with photosynthesis genes, including genes associated with light reaction, calvin cycle and CHO metabolism. Leaf L3, which is in transition from immature to mature state (Fig. 3), was highly enriched with genes necessary for building photosynthetic machinery such as tetrapyrrole biosynthesis and metabolite transporters at the envelope membrane. This developmental gradient among cassava leaves in different layers of canopy was consistent with previously published leaf development studies^32^,^33^ in monocots, in which a single developing leaf was cut evenly into several sections, indicated a conserved scheme during leaf development.

**Figure 9:**
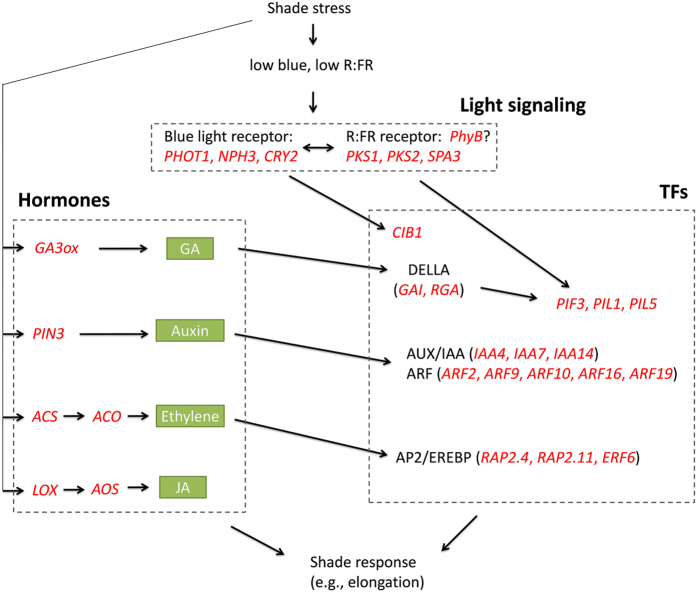
Putative gene interaction model of shade response in cassava.

Grown under shaded environments, cassava seedlings presented typical shade avoidance syndrome, e.g. significantly increased petiole length, internode length, plant height, and decreased stem diameter (Supplemental Figure S4). To discover the genes/pathways that response to the shade avoidance in cassava, we compared the transcriptome changes between shade and control conditions using mature leaves (L6-L9) to avoid the disruption from developing. We noticed a significantly decreased expression of genes that were related to calvin cycle, starch and sucrose biosynthesis, glycolysis, TCA cycle and mitochondrial electron transport, which indicated a shortage of energy supply under shaded condition, consistent with the decreased photosynthetically active radiation (PAR) and Pn (Supplemental Figure S2 and S3). To compensate for low light under shade, one reasonable behavior of cassava plant was to intend to harvest more light, since the expression of light harvesting related genes in PSI and PSII was significantly induced. In addition, the light singling perception was also changed in the shaded leaves (Figs 5 and 9). With decreased blue light under shade, the expression of PHOT1 and CRY2, two different blue light-induced receptors of phototropin and cryptochromes^34^,^35^ was induced, so do the receptor interacting genes, e.g., phototropin-interacting protein NPH3^36^ and CRY2 interacting bHLH protein CIB1^37^ (Fig. 9). Phytochrome B (phyB), the major player in shade avoidance^16^, was only slightly induced. However, phytochrome kinase substrates (PKS1 and PKS2), which can form a complex with both PHOT1 and NPH3^38^ and a phyA-105 suppressor (SPA3) were significantly induced by shade (Fig. 9, Supplemental Table S4). In addition, phytochrome interacting factors, e.g. PIF3, PIL1 (PIF3-like 1) and PIL5, which belonged to bHLH transcription factors that physically interact with phytochrome^18^,^39^,^40^, were also significantly induced in shade condition (Supplemental Table S4).

The changes of light signaling perception cross-talk with hormone mediated plant growth regulation pathways^39^,^40^. As previously reported^41^, GA3ox, which catalyzed the final step in the synthesis of bioactive GAs, was significantly induced under shade treatment. The abundance of DELLA proteins, which were the key negative regulators of GA signaling, was rapidly reduced due to accumulated GA levels^41^. However, two DELLA genes, GAI and RGA, were significantly increased in shade (Supplemental Table S4), consistent with the results of previously reported studies^42^,^43^. The further interaction between DELLA proteins and PIF family TFs (PIF3, PIL1 and PIL5) might co-regulate cell elongation related genes as reported in the hypocotyl growth^44^,^45^ of *Arabidopsis*. In addition, we also observed that the expression of GA synthesis enzyme GA20ox and GA receptors of GID1B and GID1C^46^ was greatly repressed (Supplemental Table S4), suggesting possible feedback mechanisms triggered by high levels of GA accumulation^47^,^48^. Besides GA, other hormones like auxin, ethylene and JA might also involve in shade response of cassava. For example, auxin transporter PIN3 was induced by shade, together with GRAS protein, PAT1, which encoding a polar auxin transport^18^,^41^, and TF families of Aux/IAA and ARF. Similarly, key enzymes that catalyzed the last two steps of ethylene synthesis, ACS and ACO, and most members of AP2/EREBP TF families were greatly induced by shade (Supplemental Table S4). The response of hormone metabolism, light signaling and TFs to shade indicated a complicated signaling network in shaded condition of cassava (Fig. 9).

Based on the findings of our survey and previous studies, several potential strategies could be developed to minimize the shade avoidance response in cassava. The first strategy is to regulate the expression of photoreceptor as they are light sensors for plant adaptation to different environments. Ectopic expression of phyA and phyB, two receptors of phytochromes, increased light sensitivity and finally improved yield in densely planted crops^49^,^50^. Although these two genes were not differentially expressed responding to shade in our study, they were still of great interest to examine their functions in shade response of cassava, since the genetic control of SAS can vary based on developmental time^51^. Besides, other light receptors such as PHOT1 for phototropin and CRY2 for cryptochrome, were also preferred targets to decrease shade avoidance behavior in cassava (Fig. 9). The second strategy is to attenuate the activity of positive regulators of SAS. Transcription factors, such as PIF3, PIL1 and PIL5 that can physically interact with phytochrome, have been demonstrated as positive regulators of shade avoidance^44^,^45^, in addition, their expression was significantly induced by shade in our study (Fig. 9), suggesting down-regulate the expression of these genes may be an alternative approach to minimize SAS in cassava. Last but not least, hormone regulation may be another strategy to decrease the negative effect of shade response. It has been demonstrated that auxin was a key regulator of shade response and the mutants of auxin exhibited reduced shade avoidance response to low R:FR ratio^20^. In addition to auxin, other hormones such as ethylene, GA, cytokinin and brassinosteroid were also known to play important roles in the regulation of shade response^18^,^20^. In this study, multiple genes related to ethylene, auxin and GA metabolisms were identified in response to shade stress, opening up the possibility to design a new strategy to minimize SAS in cassava.

Taken together, our study provided the first transcriptome profiling of shade response in cassava, identified the candidate genes that involved in the shade avoidance response, and offered an important resource for further investigation of the regulation of SAS pathways which can be explored to increase cassava yield by genetic improvement and intercropping management.

## Methods

### Plant material and traits measurement

Cassava (*Manihot esculenta Crantz*) cultivar, KU50, which were planted in pots (18.8 × 18.5 × 14.8 cm, height × upper diameter × bottom diameter), were used in this study. To simulate a natural shade environment that may appear in the field when intercropping newly planted cassava with other crops, half of the plants were grown under rubber trees (shade), while the other half were grown under natural sunlight (control) since March 2013 in Hainan, China. The distance between these two locations was about 100 meters, so we assume the shade was the main factor for the environmental difference.

Two months later, leaf samples, from top to the bottom of canopy, were collected for RNA-seq from shade and control respectively (Fig. 2). Each sample was pooled from six plants and repeated three times.

Physiological traits were evaluated two days before harvesting RNA-seq leaf samples. Photosynthetic parameters, including net photosynthetic rate (Pn), stomata conductance (Gs), intercellular CO_2_ concentration (Ci), transpiration rate (E) and water use efficiency (Pn/E), were measured using Li-6400 portable photosystem unit (Li-Cor Biosciences Inc., Lincoln, NB, USA) at light saturating conditions, irradiance of 1200 μmol m^−2^ s^−1^; Light spectra (including transmission and reflection) and relative light intensity were measured using HR2000 + CG high-resolution spectrometer (Ocean Optics, USA); leaf chlorophyll content (SPAD) was measured by SPAD-502 meter (Konica-Minolta, Japan); morphological parameters, including plant height, number of leaves, petiole length, internodes length and stem diameter, were measured at the same day after the leaf samples were harvested.

### RNA-Seq library preparation and sequencing

Total RNA from the leaf tissues was extracted using RNA plant reagent kits (Tiangen Company), and then purified using the TURBO DNA-free™ Kit (Ambion) to completely remove genomic DNA contamination. The integrity and quality of the total RNA were examined using a NanoDrop 1000 spectrophotometer and formaldehyde-agarose gel electrophoresis. The poly(A) RNA was isolated from purified total RNA using poly(T) oligonucleotide-attached magnetic beads (Invitrogen). Following purification, the mRNA was fragmented into small pieces using divalent cations under elevated temperatures, and the cleaved RNA fragments were reverse-transcribed into first-strand cDNA using reverse transcriptase and random primers. Second-strand cDNA synthesis was performed using DNA polymerase I and RNaseH, and the cDNA fragments were processed for end repair, a single “A” base was added, and sequences were ligated to the adapters. These products were then purified and enriched by PCR to create the final cDNA libraries and sequenced on the Illumina Hi-Seq 2500 with 51-bp lengths according to the manufacturer’s recommendations (Illumina).

### Mapping of Illumina reads and data analysis

As previously described^52^, adapters were removed from raw sequence reads using FASTX-toolkit pipeline version 0.0.13 (http://hannonlab.cshl.edu/fastx_toolkit/). Sequence quality was examined using FastQC (http://www.bioinformatics.babraham.ac.uk/projects/fastqc/). Reads were mapped to cassava genome (version 4.1) obtained from phytozome website (ftp://ftp.jgi-psf.org/pub/compgen/phytozome/v9.0/Mesculenta/) using Tophat v2.0.10 (http://tophat.cbcb.umd.edu/)^53^. Differential expressed (DE) genes were identified by Cuffdiff embed in Cufflinks pipeline v2.1.1 (http://cufflinks.cbcb.umd.edu/)^54^ and by DESeq^55^ using Bioconductor (http://www.bioconductor.org/), based on a comparison across all samples under control or shade conditions on the false discovery rate (FDR) less than 0.001. Cuffcompare in the Cufflinks package was used to identify novel isoforms, unknown intergenic transcripts, and complete matched transcripts based on the class codes.

Putative novel transcripts were predicted. In order to reduce the probability of false-positive, an in-house R-script was used to distinguish those unknown transcripts from the background signal by machine learning. Five training methods, including support vector machine, logistic regression, decision tree, random forest, and neural network, were used. The transcripts that perfect matched with the reference genome were selected. Of which, 11495 transcripts with FPKM >1.0 in all samples were selected as positive control, while 7695 with FPKM <1.0 in all samples were selected as false control. All samples reads were mixed, and four mainly parameters including transcript length, exon number, coverage, and FPKM value were considered. Area under curve (AUC), which is considered as one of most important of performance metric, was applied to select the best training method for novel transcript prediction. After that, Blast2GO^56^ was used to characterize the putative function of novel transcripts.

### Functional category enrichment and clustering analysis

Cassava loci were functionally annotated and classified into hierarchical categories using the MapMan functional classification system^57^, then significantly over-represented functional categories were identified based on Fisher’s exact test that used previously^32^. In order to define the dynamic patterns of DE genes expression along leaf development, SOTA (self-organization tree algorithm) clustering based on Pearson correlation in MEV program^58^ was used to group DE genes. The number of clusters was determined by the FOM (Figures of Merit) method. MEV was also used for gene heat-map visualization. Clusters in heat-maps were generated by arbitrarily setting a distance threshold (hierarchical clustering) when genes that were very close were grouped in a single node.

### Quantitative RT-PCR analysis

RNA-Seq results were verified by quantitative RT-PCR (qRT-PCR) using SYBR-green (TaKaRa Biotechnology Co., Ltd, Dalian, China) and ABI PRISM/TaqMan 7900 Sequence Detection System (Applied Biosystems), as described previously^59^. The primers of interested genes used in this study are provided in Supplemental Table S2. The cassava actin gene^60^ was used as an internal control. For each sample, qRT-PCR reaction was repeated three times and the relative mRNA expression level was calculated as 2^−ΔΔCT^. Correlation and significance analyses were performed using Microsoft Office Excel 2007 as before^59^.

## Additional Information

**How to cite this article**: Ding, Z. *et al*. Transcriptome response of cassava leaves under natural shade. *Sci. Rep.* **6**, 31673; doi: 10.1038/srep31673 (2016).

## Supplementary Material

Supplementary Dataset 1

Supplementary Information
