# Supplementary material for: Transcriptome response of cassava leaves under natural shade
Source: Sci Rep. 2016 Aug 19;6:31673. doi: 10.1038/srep31673 (PMC4990974; doi:10.1038/srep31673)
Supplement: Supplementary Information [file srep31673-s2.doc]

**Supplemental Information**

**Transcriptome response of cassava leaves under natural shade**

Zehong Ding^$1^, Yang Zhang^$2,3^, Yi Xiao^4^, Fangfang Liu^5^, Minghui Wang^6^, Xinguang Zhu^4^, Peng Liu^5^, Qi Sun^6^, Wenquan Wang^1^, Ming Peng^1^, Tom Brutnell^7^, Pinghua Li*^8^

^1^The Institute of Tropical Bioscience and Biotechnology (ITBB), Chinese Academy of Tropical Agricultural Sciences (CATAS), Haikou, Hainan 571101, China

^2^Center for Plant Science Innovation, University of Nebraska-Lincoln, Nebraska, USA

^3^Department of Agronomy and Horticulture, University of Nebraska-Lincoln, Nebraska, USA

^4^CAS-Key laboratory of Computational Biology, CAS-MPG Partner Institute for Computational Biology, Shanghai Institutes for Biological Sciences, CAS, Shanghai 200031, China

^5^Department of Statistics, Iowa State University, Ames, Iowa 50011, USA

^6^Computational Biology Service Unit, Life Sciences Core Laboratories Center, Cornell University, Ithaca, New York 14850, USA.

^7^Donald Danforth Plant Science Center, St. Louis, Missouri 63132, USA

^8^College of Agronomic Sciences, Shandong Agricultural University, Tai’an, Shandong 271018, China.

^$^These authors contributed equally to this work.

Correspondence should be addressed to PH Li (pinghuali@sdau.edu.cn)


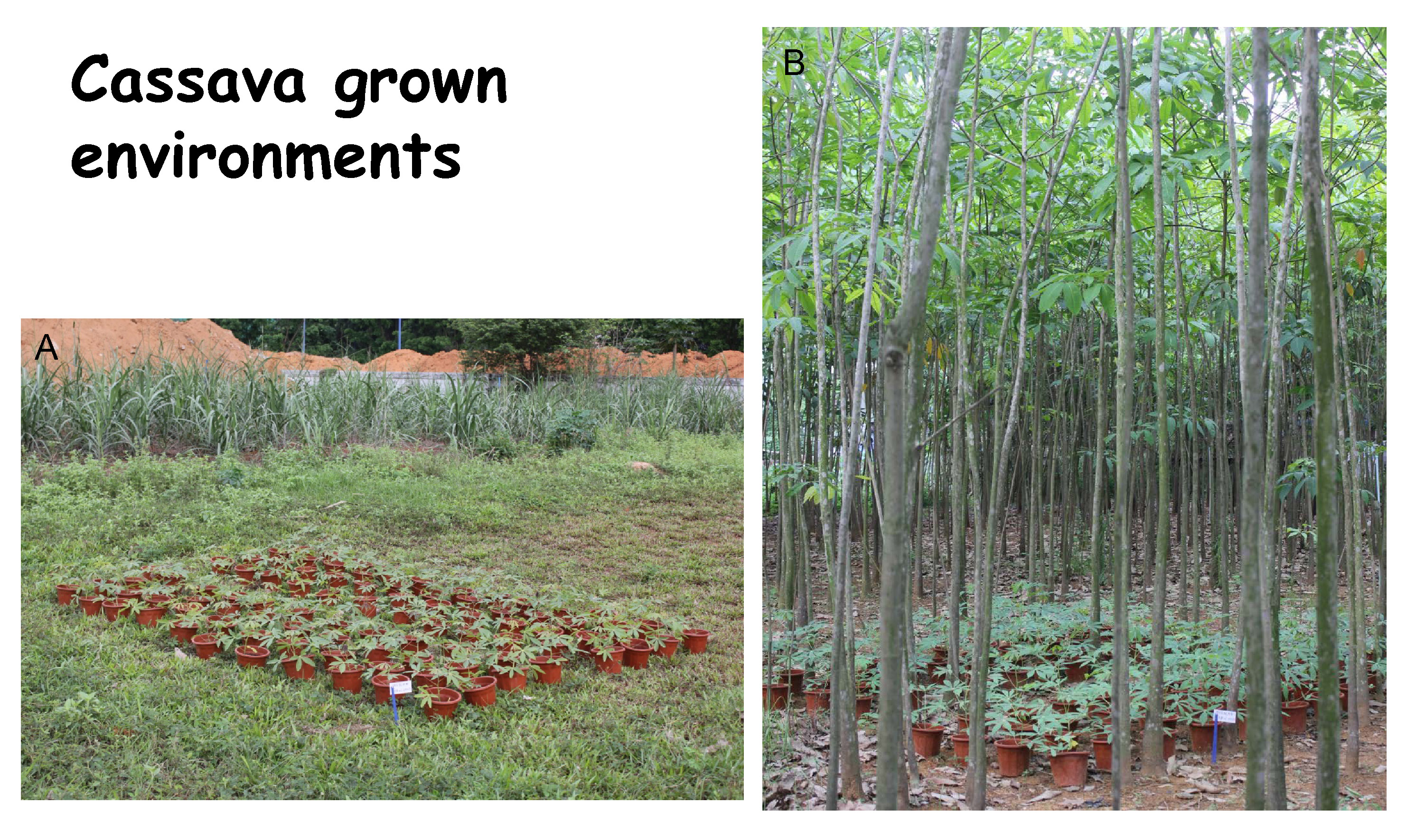


**Figure S1 Grown environments of cassava in full sunlight (A) and natural shade (B) conditions, respectively.**


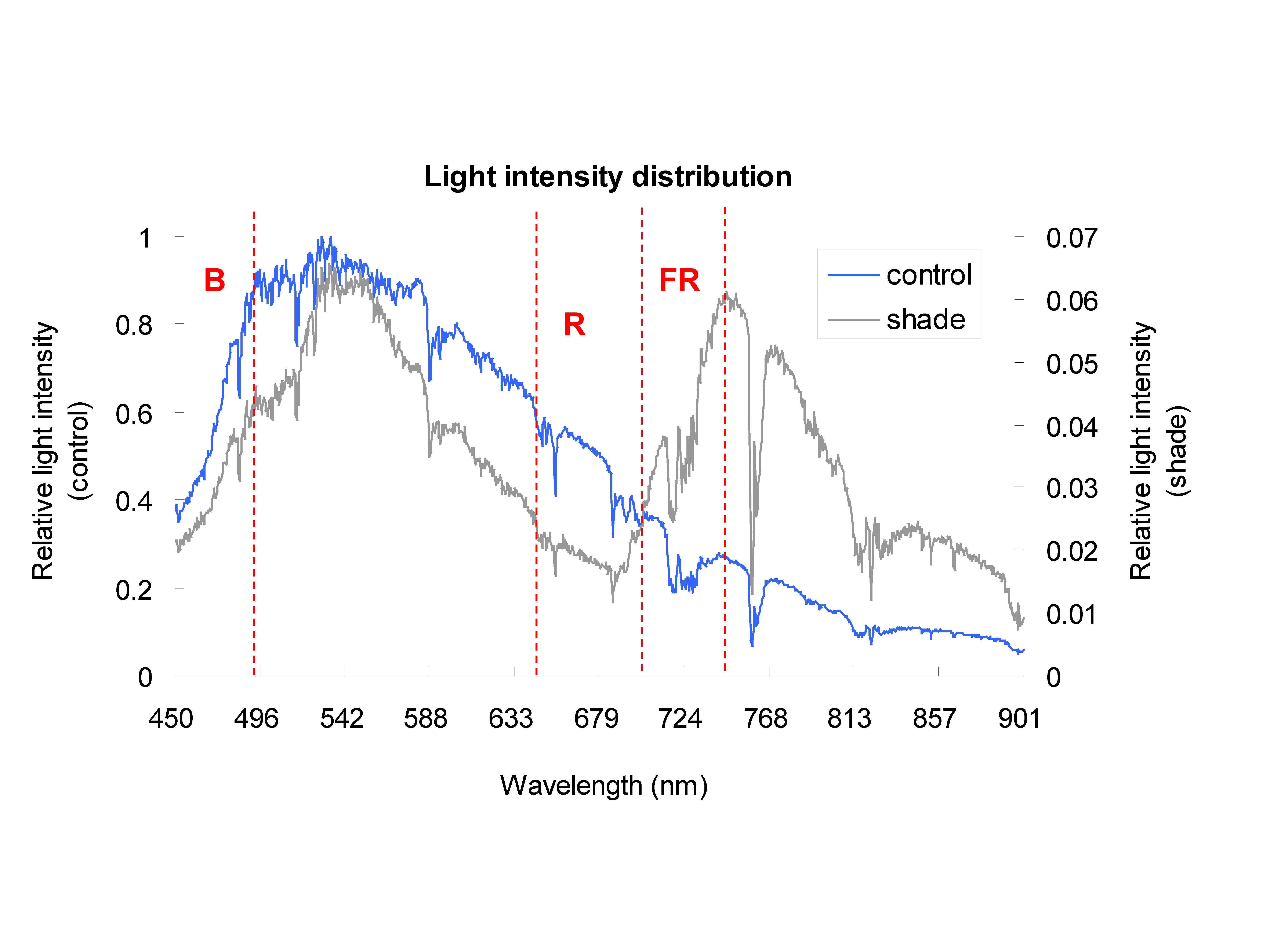


**Figure S2 Distribution of relative light intensity in control and shade conditions, respectively.**


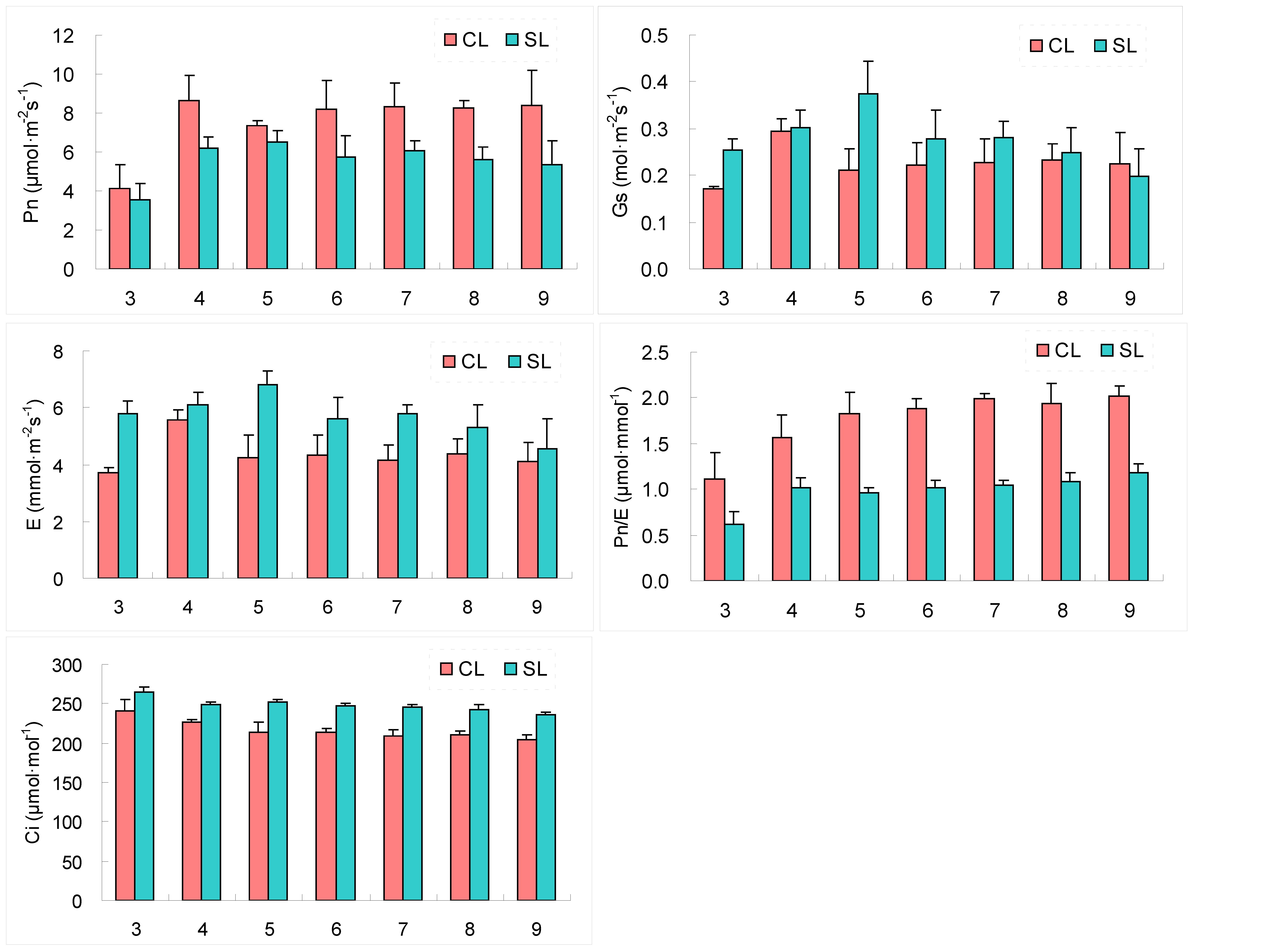


**Figure S3 Photosynthetic parameters of expanded leaves (L3-L9) in control and shade conditions, respectively.**

Photosynthetic parameters, including net photosynthetic rate (Pn), stomata conductance (Gs), intercellular CO_2_ concentration (Ci), transpiration rate (E) and water use efficiency (Pn/E), were investigated.


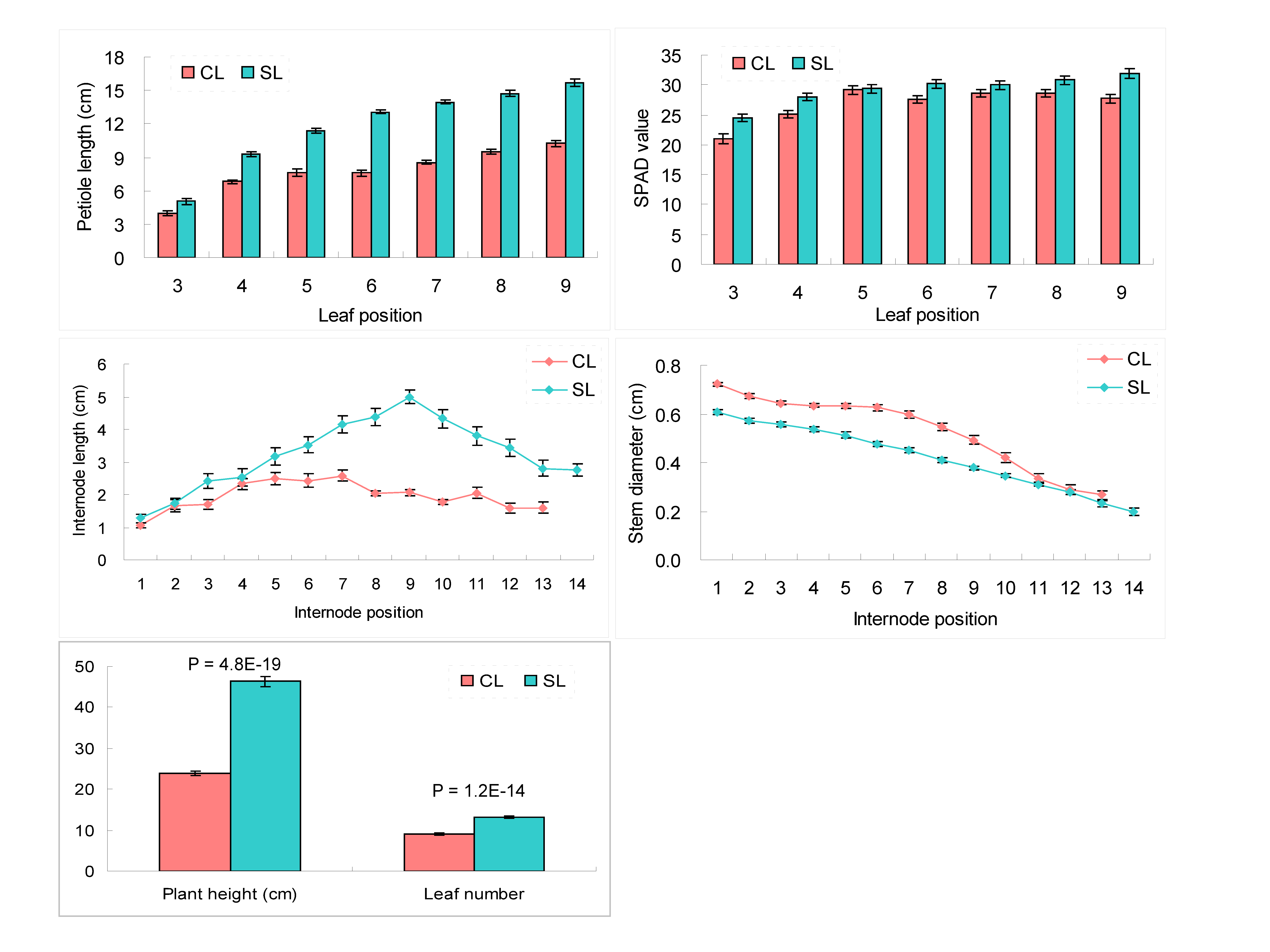


**Figure S4 Growth changes of cassava in response to shade.**


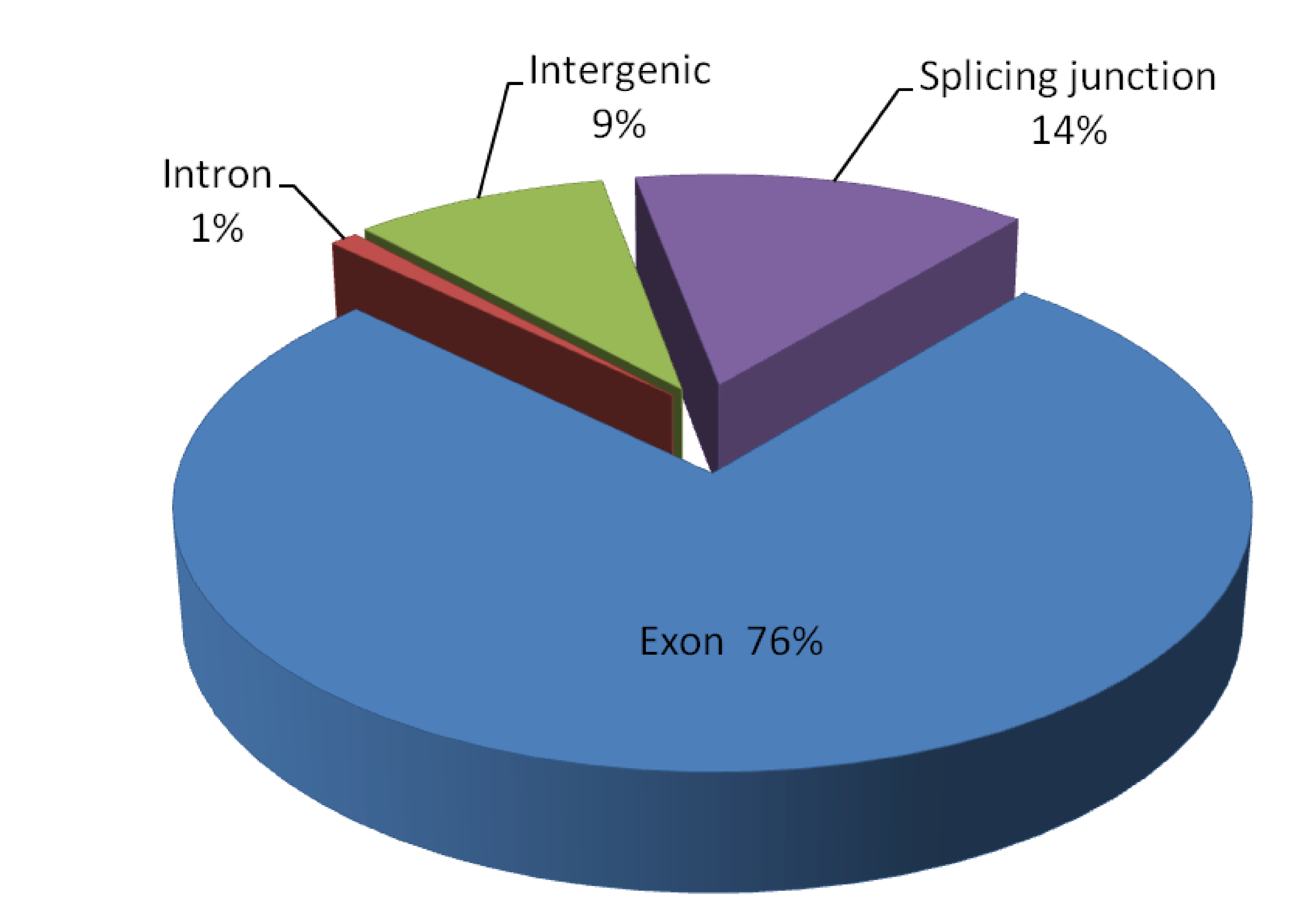


**Figure S5 Reads distribution of developmental cassava leaves.**


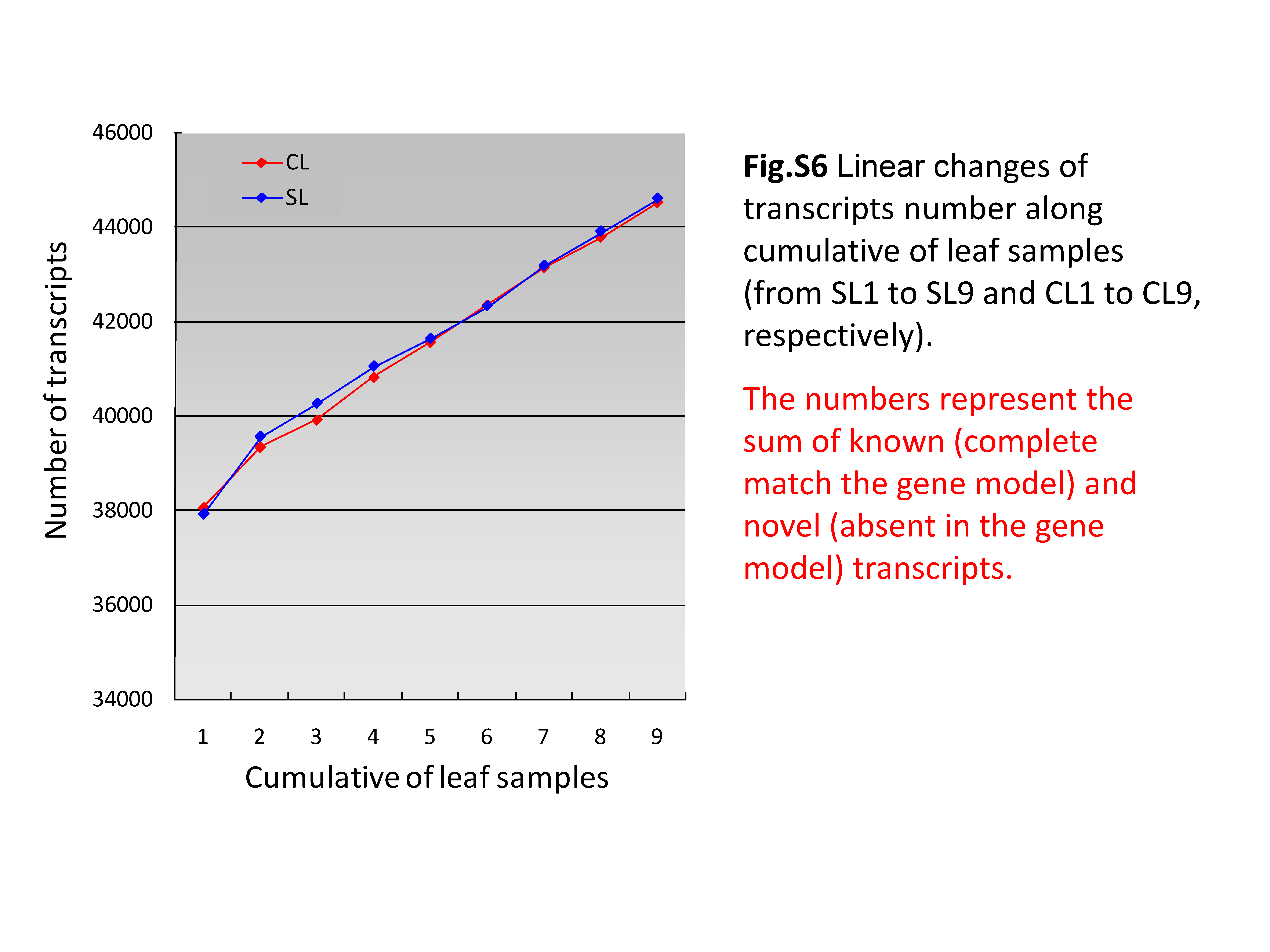


**Figure S6 Linear changes of transcripts number along cumulative of leaf samples.** Leaves from young to old: CL3-CL9 for control and SL3-SL9 for shade. The numbers represent the sum of known (complete match the gene model) and novel (absent in the gene model) transcripts.


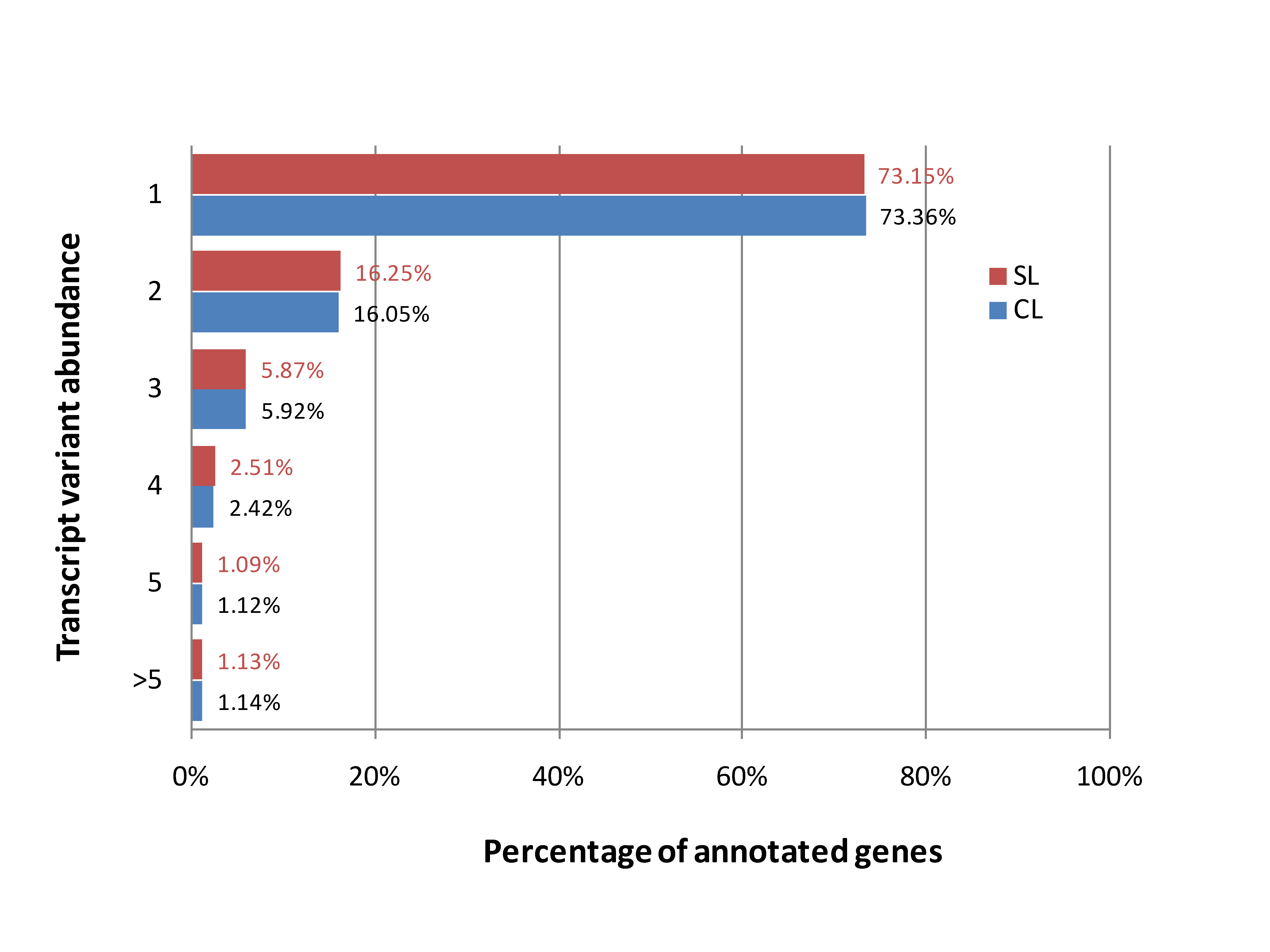


**Figure S7 Distribution of genes with a given number of transcript variants.**


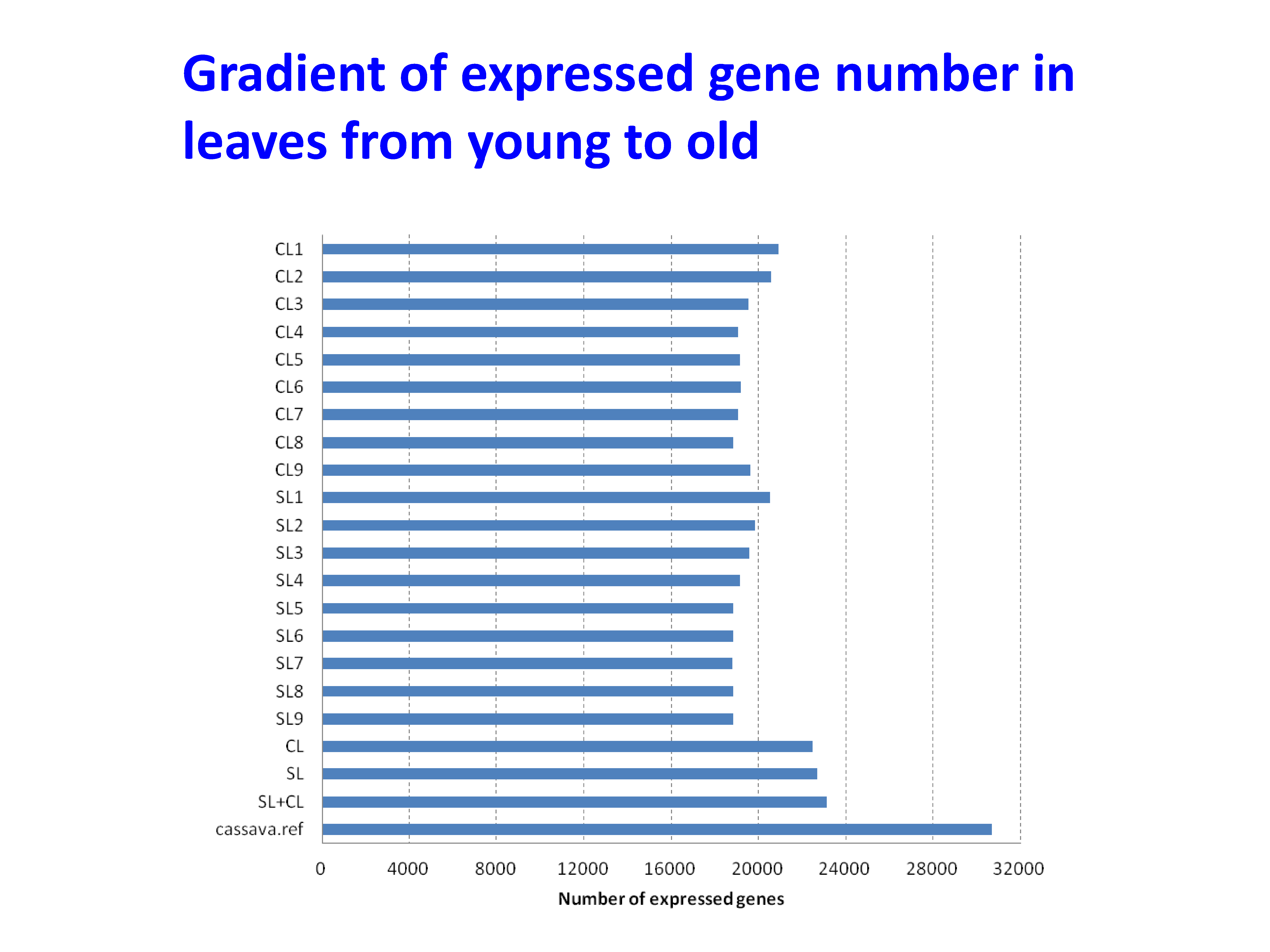


**Figure S8 Gradient of expressed gene number in leaves from young to old.**


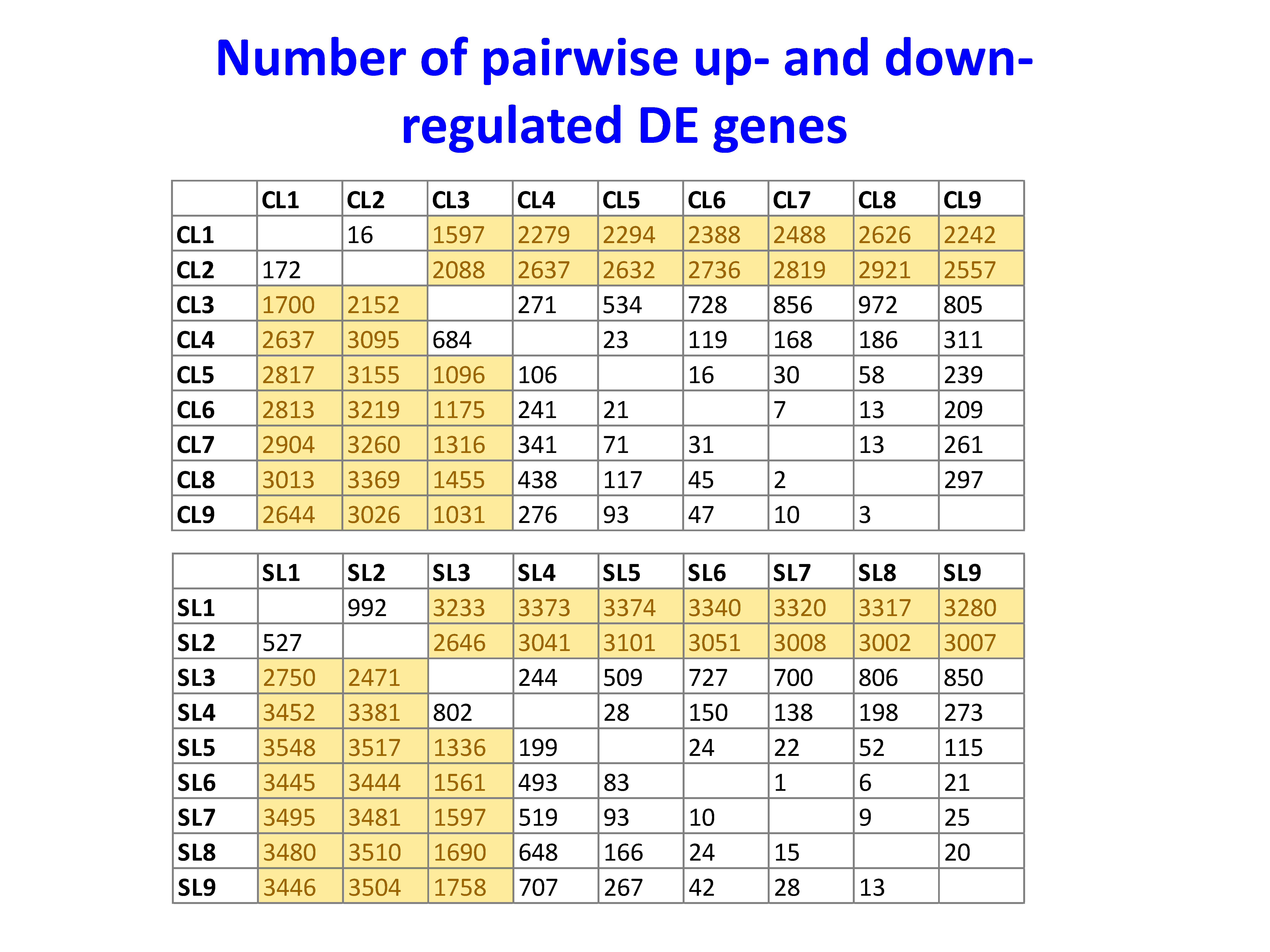


**Figure S9 Number of pairwise up- and down-regulated DE genes.** Numbers in the matrix represent the number of up/down regulated genes comparing the higher number-index leaf with the lower number-index leaf. Numbers above/down diagonal represent up/down regulated genes number, respectively.


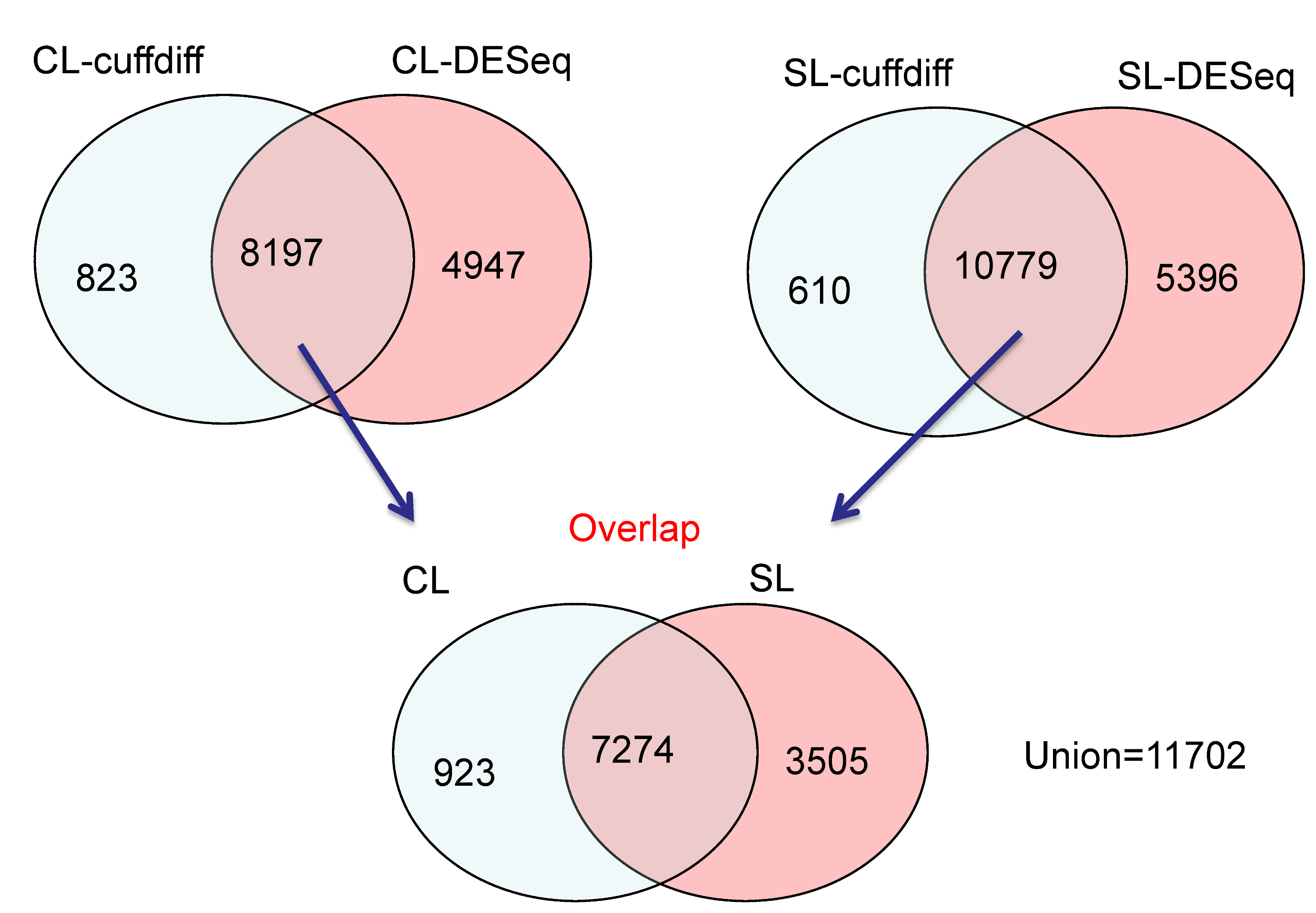


**Figure S10 Summary of DE genes comparison.**


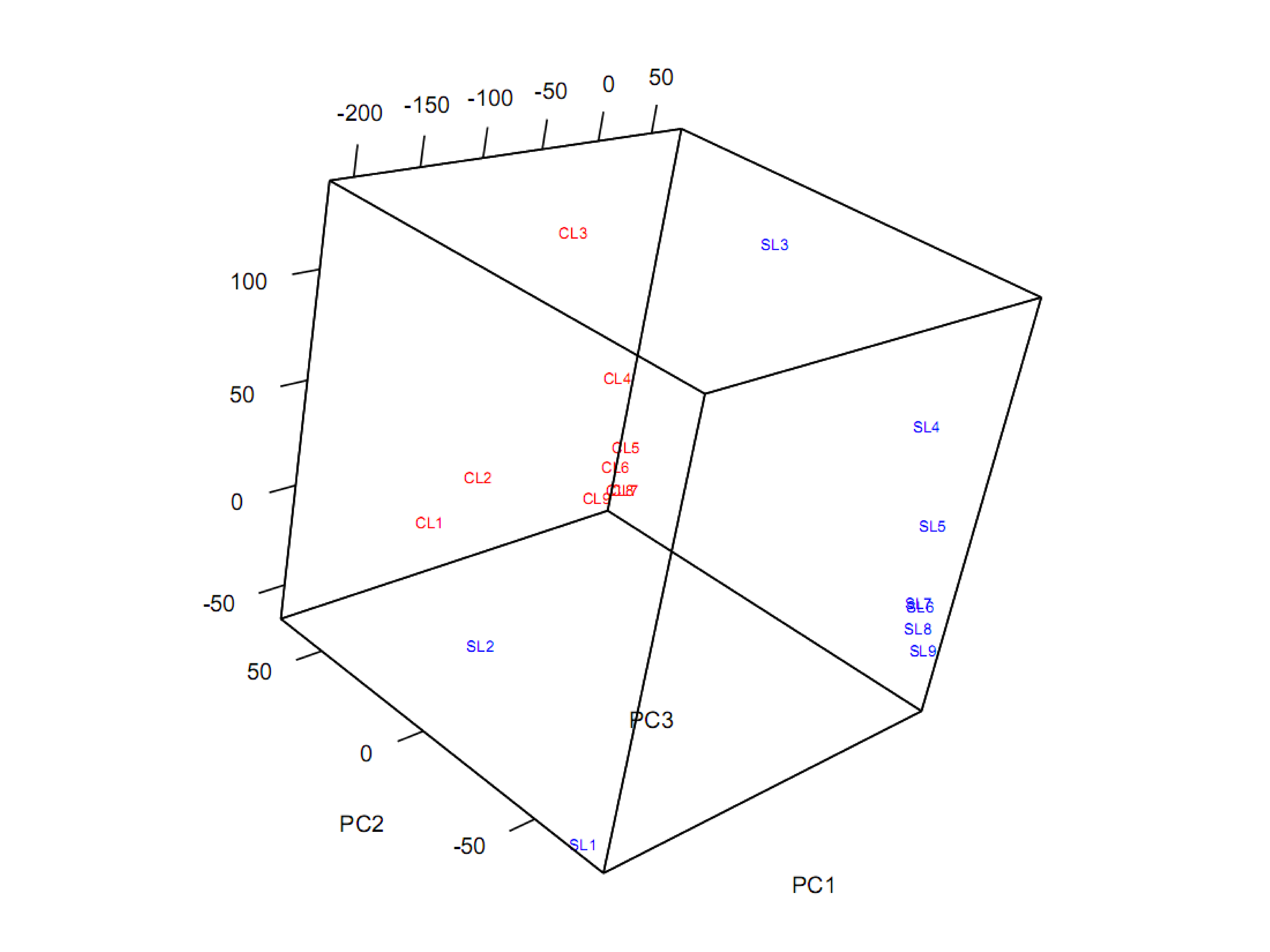


**Figure S11 Principal component analysis (PCA) of the leaf samples used in this study.**
